# Efficacy and effect on lipid profiles of switching to ainuovirine-based regimen versus continuing efavirenz-based regimen in people with HIV-1: 24-week results from a real-world, retrospective, multi-center cohort study

**DOI:** 10.1128/aac.01668-23

**Published:** 2024-03-14

**Authors:** Chunmei Wang, Xiaoli Yu, Yingchun Ke, Yanhua Fu, Yanhe Luo, Ying Li, Yanmei Bi, Xingqiong Chen, Linghua Li, Xiuhong Zhao, Zhong Chen

**Affiliations:** 1 Department of Dermatology, Shandong Public Health Clinical Center, Shandong University, Jinan, Shandong, China; 2 Department of Infection and Immunology with Chinese Integrative Medicine, Wuhan Jinyintan Hospital, Tongji Medical College of Huazhong University of Science and Technology, Wuhan, China; 3 Infectious Disease Center, Guangzhou Eighth People’s Hospital, Guangzhou Medical University, Guangzhou, Guangdong, China; 4 Department of Infectious Disease, GuiYang Public Health Clinical Center, Guiyang, Guizhou, China; 5 Department of Infection and Immunology, The First Hospital of Changsha City, Xiangya School of Medicine of Central South University, Changsha, Hunan, China; 6 Department of Outpatient, Yunnan Provincial Infectious Disease Hospital, Kunming, Yunnan, China; IrsiCaixa Institut de Recerca de la Sida, Barcelona, Spain

**Keywords:** ainuovirine, lipid, efavirenz, HIV-1 infection, treatment-experienced

## Abstract

Ainuovirine (ANV), a novel non-nucleoside reverse-transcriptase inhibitor (NNRTI), was approved in China in 2021. In a previous randomized phase 3 trial, ANV demonstrated non-inferior efficacy relative to efavirenz (EFV) and was associated with lower rates of dyslipidemia. In this study, we aimed to explore lipid changes in treatment-experienced people with human immunodeficiency virus (HIV)-1 (PWH) switching to ANV from EFV in real world. At week 24, 96.65% of patients in the ANV group and 93.25% in the EFV group had HIV-1 RNA levels below the limit of quantification (LOQ). Median changes from baseline in CD4 +T cell counts (37.0 vs 36.0 cells/µL, *P* = 0.886) and CD4+/CD8 +ratio (0.03 vs 0.10, *P* = 0.360) were similar between the two groups. The ANV group was superior to the EFV group in mean changes in total cholesterol (TC, −0.06 vs 0.26 mmol/L, *P* = 0.006), triglyceride (TG, −0.6 vs 0.14 mmol/L, *P* < 0.001), high-density lipoprotein cholesterol (HDL-C, 0.09 vs 0.08 mmol/L, *P* = 0.006), and low-density lipoprotein cholesterol (LDL-C, −0.18 vs 0.29 mmol/L, *P* < 0.001) at week 24. We also observed that a higher proportion of patients demonstrated improved TC (13.55% vs 4.45%, *P* = 0.015) or LDL-C (12.93% vs 6.89%, *P* = 0.017), and a lower proportion of patients showed worsened LDL-C (5.57% vs 13.52%, *P* = 0.017) with ANV than with EFV at week 24. In conclusion, we observed good efficacy and favorable changes in lipids in switching to ANV from EFV in treatment-experienced PWH in real world, indicating a promising switching option for PWH who may be more prone to metabolic or cardiovascular diseases.

## INTRODUCTION

The introduction of highly active antiretroviral therapy (HAART) in 1996 and 1997 led to marked reductions in human immunodeficiency virus (HIV)/acquired immune deficiency syndrome (AIDS)-related mortality and morbidity rates and increased the life expectancy of people with HIV (1, 2). However, fewer AIDS-related deaths and an aging cohort have resulted in an increase in the proportion of people with HIV dying from non-AIDS illnesses, such as cardiovascular diseases (CVD), liver disease, and non-AIDS malignancies (3, 4). Among those, CVD has become an increasingly common cause of death in people with HIV (5). People with HIV are twice as likely to develop CVD as individuals without HIV, and the global burden of HIV-associated CVD tripled between 1990 and 2015 (6). People with HIV exhibit multiple known CVD risk factors notable for dyslipidemia (7). Dyslipidemia is highly prevalent among people with HIV (8). Intrinsically, HIV infection itself is associated with abnormalities in lipid metabolism (9). Persistent inflammation and immune activation in HIV infection can alter lipid and metabolic profiles (10). Furthermore, dyslipidemia is a major long-term adverse effect of current antiretroviral therapy (ART) regimens. Several studies have shown that ART is associated with dyslipidemia (11–13), and distinct ART regimens appear to promote different modifications in lipid metabolism (14). In people with HIV, ART regimens showed a 63.8% prevalence of dyslipidemia (15). In short, the prevalence of dyslipidemia, whether genetically determined or influenced by ART regimen or HIV infection, is consistently higher in HIV-infected groups (16). Dyslipidemia is characterized by elevated levels of total cholesterol (TC), low-density lipoprotein cholesterol (LDL-C), triglycerides (TG), or decreased high-density lipoprotein cholesterol (HDL-C) levels (17). LDL-C is an important causal risk factor for atherosclerotic CVD (18). Moreover, dyslipidemia is usually associated with hypertension, type 2 diabetes, and metabolic syndrome (19, 20). It is important to monitor, evaluate, and manage dyslipidemia in people with HIV.

Efavirenz (EFV), a non-nucleoside reverse-transcriptase inhibitor (NNRTI), is one of the most commonly prescribed antiretroviral medications worldwide (21) and is included in the World Health Organization’s preferred first-line ART regimens for people with HIV (22). EFV is also widely used in China since its inclusion as a nationally approved free anti-AIDS drug (23). A Chinese retrospective study including 7,623 people with HIV showed that EFV-based regimens were the most commonly selected first-line ART, accounting for 93.1% of the study population (24). Ainuovirine (ANV), a new generation of NNRTI independently developed in China, was approved in 2021. A randomized phase 3 trial indicated that the antiviral efficacy of ANV was non-inferior to EFV when combined with two nucleoside reverse transcriptase inhibitors (NRTIs), and treatment-emergent adverse events (TEAEs) in ANV-treated participants were less frequent regarding liver toxicity, dyslipidemia, neuropsychiatric symptoms, and rash compared with the EFV group (25). Given the strict inclusion criteria in the phase 3 trial, it was important to further understand the antiviral efficacy and effects on the lipid profile of ANV in real-world patients.

## MATERIALS AND METHODS

### Study design

A real-world, retrospective, multi-center controlled cohort study was conducted to evaluate the antiviral efficacy and effect on the lipid profile of switching to ANV+2 NRTIs in people with HIV-1 who were previously treated with EFV+2 NRTIs. The study was conducted at six sites in China namely: Jinan, Wuhan, Changsha, Guangzhou, Guiyang, and Kunming (Table S1).

Eligible participants were diagnosed with HIV-1, aged 18 years or older, had received antiretroviral therapy, and achieved virological suppression, which was defined as an HIV-1 RNA level below the limit of quantification (LOQ) (see Table S2 for LOQ criteria). In addition, the study also included patients with HIV-1 RNA levels above the LOQ who were considered appropriate for switching in the ANV group and without treatment failure in the EFV group at the physician’s discretion. Participants with severe metabolic abnormalities, cardiovascular diseases, and major neurological and psychiatric diseases were excluded. Women who were pregnant or breastfeeding and women of childbearing age or whose partners were unable to take effective contraception measures were not included either.

The ANV group was selected through the HIV real-world research platform (i-Study), of which the participants all signed the informed consent form. This group of patients had been receiving the EFV-based regimen, which included lamivudine (3TC) and tenofovir disoproxil fumarate (TDF) combined with EFV. Then, these patients switched to the ANV-based regimen, which was a once daily triple-drug therapy, either included with three drugs of ANV (75 mg/tablet ×2 tablets) +3 TC (300 mg ×1 tablet) +TDF (300 mg ×1 tablet) or a fixed-dose compound tablet of ANV 150 mg/3TC 300 mg/TDF 300 mg (Ainuovirine, Lamivudine and Tenofovir Disoproxil Fumarate Tablets, provided by Aidea Pharma, Jiangsu, China) and continued to take the regimen for at least 24 weeks. Drug switching was due to intolerance to the EFV-based regimen or at the physician’s discretion. The EFV group was screened from the National Free Antiretroviral Treatment Program Database in China. This group of patients had been receiving the EFV-based regimen, a once daily triple-drug therapy including EFV 600 mg or 400 mg (×1 tablet) +3 TC 300 mg (×1 tablet) +TDF 300 mg (×1 tablet) and continued to take it for at least 24 weeks from baseline.

### Assessment

Patient data were collected from medical records. All participants were required to have a lipid profile (TC, TG, HDL-C, LDL-C) determined at baseline and at 12 and 24 weeks. Other laboratory test results, such as HIV-1 RNA level, CD4^+^ T-cell count, and CD4^+^/CD8^+^ ratio, were also collected. Fasting lipid profile, CD4^+^, and CD8^+^ T-cell counts were measured by Cobas C702 Chemistry Analyzer (Roche Diagnostics, Mannheim, Germany) at all sites, following the manufacturer’s recommended procedures. HIV-1 RNA levels were measured using NEPG Automatic HIV-1 RNA Quantitative Test Kit (Northeast Pharm Group Liaoning Biomedicine Co., LTD, Liaoning, China; LOQ = 100 copies/mL) at Wuhan Jinyintan Hospital, and Xpert HIV-1 Viral Load assay (Cepheid, California, USA; LOQ = 20 copies/mL) was used at other sites. All other clinical measurements were routinely conducted in the Clinical Laboratory Department at each site. An abnormal lipid profile was defined according to the criteria outlined in the 2023 Chinese Guidelines for Lipid Management, with thresholds set as follows: TC ≥5.2 mmol/L, TG ≥1.7 mmol/L, HDL-C <1.0 mmol/L, or LDL-C ≥3.4 mmol/L (26).

The primary endpoints were efficacy on viral suppression (HIV-1 RNA levels below the LOQ, change in the CD4^+^ T-cell count and CD4^+^/CD8^+^ ratio) and changes in lipid profile [TC, TG, HDL-C, LDL-C, and log (TG/HDL-C)] at week 24. The secondary endpoints were efficacy on viral suppression and changes in lipid profile at week 12.

### Statistical analysis

Propensity score weighting using overlap weights was proven to be an appropriate and feasible method to adjust for confounding factors (27). In this study, baseline information, such as age, sex, antiviral treatment duration, WHO stage, comorbidities (hypertension, diabetes, etc.), CD4^+^ T-cell count, and body weight (BMI) were used to calculate the propensity scores. For all variables, overlap weights were calculated based on propensity scores for weighted analysis. Continuous variables are presented as the mean (standard deviation), and an independent samples *t* test was used for intergroup comparisons. Categorical variables are reported as numbers (percentages), and the χ^2^ test was used for intergroup comparisons. The mean and 95% confidence intervals (CIs) were used to summarize the lipid profile changes from baseline at weeks 12 and 24, and the analysis of covariance was used to adjust the baseline levels for intergroup comparisons. All analyses were performed using R 4.2.2 and SAS Version 9.4.

## RESULTS

### Patient baseline characteristics

We retrospectively identified 318 patients eligible for the ANV group and 460 patients for the EFV group. Table 1 summarizes the unweighted and overlap weighted patient baseline characteristics and the standardized mean differences (SMDs) for each variable. After overlap weighting, the baseline characteristics between the two groups were well balanced with SMD <0.1 (Table 1). Both groups had 157 patients, the mean age was 40, 90.9% of them were male, and 8.0% of the study population had comorbidities (i.e., hypertension, diabetes). The average duration of antiviral treatment was 34.8 weeks in both groups. At baseline, the proportion of HIV RNA levels below the LOQ was 88.9% in the ANV group and 94.3% in the EFV group (*P* = 0.012). The mean CD4^+^ T-cell count in both groups was 510.8 cells/µL (*P* = 1.000), and the mean CD4^+^/CD8^+^ ratio was 0.68 in the ANV group and 0.69 in the EFV group (*P* = 0.655).

**TABLE 1:** Baseline characteristics^*a*^

	Unweighted				Overlap weighting on propensity score^*b*^			
	ANV group	EFV group	*P*	SMD	ANV group	EFV group	*P*	SMD
Patients, *n*	318	460			157.41	157.41		
Age, mean (SD), y	41.87 (12.88)	36.70 (13.28)	<0.001	0.395	40.29 (12.69)	40.29 (14.47)	1.000	<0.001
Sex, *n* (%)								
Male	288 (90.6)	423 (92.0)	0.583	0.049	143.0 (90.9)	143.0 (90.9)	1.000	<0.001
Female	30 (9.4)	37 (8.0)			14.4 (9.1)	14.4 (9.1)		
Treatment duration, mean (SD), wk	50.23 (37.16)	28.30 (17.88)	<0.001	0.752	34.83 (27.28)	34.83 (20.90)	1.000	<0.001
WHO stage for HIV/AIDS, *n* (%)								
Stage 1	196 (61.6)	291 (63.3)	0.353	0.132	93.7 (59.5)	93.7 (59.5)	1.000	<0.001
Stage 2	77 (24.2)	92 (20.0)			39.6 (25.1)	39.6 (25.1)		
Stage 3	26 (8.2)	51 (11.1)			14.6 (9.3)	14.6 (9.3)		
Stage 4	19 (6.0)	26 (5.7)			9.5 (6.0)	9.5 (6.0)		
Comorbidities, *n* (%)								
None	285 (89.6)	439 (95.4)	0.003	0.222	144.8 (92.0)	144.8 (92.0)	1.000	<0.001
Yes	33 (10.4)	21 (4.6)			12.6 (8.0)	12.6 (8.0)		
CD4^+^ T-cell count, mean (SD), cells/μL	529.39 (273.13)	519.93 (240.35)	0.610	0.037	510.84 (268.47)	510.84 (232.72)	1.000	<0.001
CD4^+^/CD8^+^ ratio, mean (SD)	0.73 (0.43)	0.68 (0.36)	0.176	0.112	0.68 (0.40)	0.69 (0.37)	0.655	0.040
HIV-1 RNA, *n* (%)								
Below the LOQ	290 (91.2)	396 (93.4)	0.326	0.083	140.0 (88.9)	140.5 (94.3)	0.012	0.195
Above the LOQ	28 (8.8)	28 (6.6)			17.4 (11.1)	8.5 (5.7)		
Height, mean (SD), cm	171.45 (7.28)	173.79 (7.62)	<0.001	0.314	171.69 (7.25)	173.33 (7.71)	0.006	0.219
Weight, mean (SD), kg	67.54 (11.03)	68.92 (12.01)	0.104	0.120	67.75 (11.25)	67.75 (11.89)	1.000	<0.001
BMI, mean (SD)	22.89 (2.92)	22.77 (3.36)	0.6	0.039	22.90 (2.98)	22.49 (3.30)	0.106	0.128
<18.5, *n* (%)	25 (7.9)	42 (9.1)	0.574	0.103	13.1 (8.3)	15.7 (9.9)	0.35	0.144
18.5**–**24, *n* (%)	178 (56.0)	268 (58.3)			88.1 (55.9)	95.7 (60.8)		
24–28, *n* (%)	103 (32.4)	129 (28.0)			50.3 (32.0)	40.5 (25.8)		
≥28, *n* (%)	12 (3.8)	21 (4.6)			5.9 (3.8)	5.5 (3.5)		
SBP, mean (SD), mm Hg	123.28 (10.51)	123.93 (7.29)	0.345	0.072	123.12 (10.40)	124.08 (7.22)	0.216	0.107
DBP, mean (SD), mm Hg	79.18 (8.05)	77.52 (8.08)	0.008	0.206	79.02 (7.95)	77.37 (7.92)	0.013	0.207
RBC, mean (SD), ×10^12^ /L	4.59 (0.60)	4.77 (0.52)	<0.001	0.321	4.65 (0.59)	4.74 (0.53)	0.052	0.158
Hb, mean (SD), g/L	149.68 (17.31)	152.39 (17.71)	0.035	0.155	150.73 (16.48)	151.13 (17.93)	0.760	0.024
PLT, mean (SD), ×10^9^ /L	227.90 (63.86)	243.93 (60.81)	<0.001	0.257	226.26 (61.97)	242.16 (64.32)	0.002	0.252
WBC, mean (SD), ×10^9^ /L	6.03 (1.68)	6.37 (1.69)	0.006	0.200	6.05 (1.66)	6.35 (1.74)	0.026	0.180
ALT, mean (SD), U/L	45.23 (47.82)	34.44 (21.67)	<0.001	0.291	46.84 (53.32)	33.46 (20.67)	<0.001	0.331
AST, mean (SD), U/L	35.95 (27.46)	30.39 (15.36)	<0.001	0.250	36.93 (29.79)	30.96 (13.88)	0.004	0.257
TB, mean (SD), μmol/L	10.22 (4.54)	8.70 (3.68)	<0.001	0.368	10.13 (4.47)	8.88 (3.43)	<0.001	0.314
DB, mean (SD), μmol/L	3.70 (1.64)	3.66 (1.60)	0.755	0.023	3.67 (1.58)	3.63 (1.59)	0.714	0.029
BG, mean (SD), mmol/L	5.82 (1.68)	7.28 (31.69)	0.437	0.065	5.85 (1.80)	7.06 (29.29)	0.406	0.058
SUA, mean (SD), μmol/L	356.31 (97.64)	341.86 (96.60)	0.042	0.149	361.19 (100.02)	343.32 (97.00)	0.026	0.181
SCr, mean (SD), mmol/L	71.96 (14.86)	72.83 (11.80)	0.367	0.065	71.20 (15.23)	72.68 (12.21)	0.203	0.107
BUN, mean (SD), mmol/L	4.97 (1.15)	4.55 (1.34)	<0.001	0.336	4.93 (1.13)	4.62 (1.36)	0.002	0.245
TC, mean (SD), mmol/L	4.65 (1.02)	4.28 (1.18)	<0.001	0.334	4.59 (1.04)	4.29 (1.07)	<0.001	0.283
Normal, *n* (%)	232 (73.0)	395 (85.9)	<0.001	0.324	119.0 (75.6)	134.2 (85.3)	0.002	0.245
Abnormal, *n* (%)	86 (27.0)	65 (14.1)			38.4 (24.4)	23.2 (14.7)		
TG, mean (SD), mmol/L	2.47 (2.13)	1.76 (1.32)	<0.001	0.399	2.46 (2.11)	1.75 (1.26)	<0.001	0.408
Normal, *n* (%)	139 (43.7)	282 (61.3)	<0.001	0.358	72.7 (46.2)	95.6 (60.7)	<0.001	0.295
Abnormal, *n* (%)	179 (56.3)	178 (38.7)			84.7 (53.8)	61.8 (39.3)		
HDL-C, mean (SD), mmol/L	1.06 (0.31)	1.14 (0.45)	0.004	0.215	1.05 (0.30)	1.15 (0.50)	0.002	0.254
Normal, *n* (%)	175 (55.0)	308 (67.0)	0.001	0.246	84.6 (53.8)	105.4 (67.0)	0.001	0.273
Abnormal, *n* (%)	143 (45.0)	152 (33.0)			72.8 (46.2)	52.0 (33.0)		
LDL-C, mean (SD), mmol/L	2.80 (0.95)	2.43 (0.87)	<0.001	0.401	2.74 (0.95)	2.45 (0.84)	<0.001	0.325
Normal, *n* (%)	246 (77.4)	402 (87.4)	<0.001	0.266	125.6 (79.8)	137.9 (87.6)	0.006	0.213
Abnormal, *n* (%)	72 (22.6)	58 (12.6)			31.8 (20.2)	19.5 (12.4)		
Log(TG/HDL-C), mean (SD)	0.27 (0.36)	0.12 (0.32)	<0.001	0.448	0.28 (0.37)	0.12 (0.31)	<0.001	0.441

^*a*^ ALT, alanine transaminase; ANV, ainuovirine; AST, aspartate aminotransferase; BG, blood glucose; BUN, blood urea nitrogen; DB, direct bilirubin; DBP, diastolic blood pressure; EFV, efavirenz; Hb, hemoglobin; HDL-C, high density lipoprotein cholesterol; LDL-C, low density lipoprotein cholesterol; LOQ, limit of quantification; PLT, platelet; RBC, red blood cell; SBP, systolic blood pressure; SCr, serum creatinine; SD, standard deviation; SMD, standardized mean differences; SUA, serum uric acid; TB, total bilirubin; TC, total cholesterol; TG, triglyceride; WBC, white blood cell.

^*b*^ Covariates adjusted by propensity scores include age, sex, antiviral treatment duration, WHO stage, comorbidities, CD4^+^ count, and baseline weight. Propensity score weighting using overlap weights.

### Efficacy

At week 24, there were numerically more patients with HIV-1 RNA levels below the limit of quantification (LOQ) in the ANV group (baseline 88.9% to 96.65%) than in the EFV group (baseline 94.3% to 93.25%), although there were no significant differences between groups (Table 2).

**TABLE 2:** Efficacy outcomes at week 24^*a*^

	Overall	ANV group (*n* = 318)	EFV group (*n* = 460)	*P* value
HIV-1 RNA				
Patients (missing data), *n*	461 (317)	318 (0)	143 (317)	
Below the LOQ, %	95.83%	96.65%	93.25%	^*b*^
Above the LOQ, %	4.17%	3.35%	6.75%	
CD4^+^ T cell count change from baseline				
Patients (missing data), *n*	522 (256)	310 (8)	212 (248)	
Median (IQR), cells/μL	37.00 (−10.00 to 121.00)	37.00 (3.00–99.00)^*c*^	36.00 (−51.00 to 178.00)^*c*^	0.886
CD4^+^/CD8^+^ ratio change from baseline				
Patients (missing data), *n*	401 (377)	236 (82)	165 (295)	
Median (IQR)	0.03 (−0.02 to 0.10)	0.03 (−0.01 to 0.10)^*c*^	0.10 (−0.10 to 0.20)^*c*^	0.360

^*a*^ ANV, ainuovirine; EFV, efavirenz; IQR, interquartile range; LOQ, limit of quantification; SD, standard deviation. The number of patients and missing data were unweighted, and the remaining results were obtained through weighted analysis on propensity score. Paired *t* test (week 24 vs baseline) was used for intragroup comparison, and analysis of covariance was used for intergroup comparison of changes from baseline.

^*b*^ *P* value is not valid for intergroup comparison.

^*c*^ *P* < 0.05 for intragroup comparison.

In primary analysis, similar changes from baseline in CD4^+^ T-cell count and CD4^+^/CD8^+^ ratio were observed in the two groups at week 24 (Table 2). The median increases from baseline in CD4^+^ T-cell count were 37.0 cells/µL [interquartile range (IQR), 3.0 to 99.0] in the ANV group and 36.0 cells/µL (IQR, −51.0 to 178.0) in the EFV group, with no significant differences between groups (*P* = 0.886). The median increases in the CD4^+^/CD8^+^ ratio were 0.03 (IQR, −0.01 to 0.10) with ANV and 0.10 (IQR, −0.10 to 0.20) with EFV, and no significant differences were observed between the two groups (*P* = 0.360). These data demonstrated that the efficacy of switching to the ANV-based regimen was comparable to that of continuing EFV-based regimen at week 24.

### Changes in lipid profile

At baseline, the mean TC, TG, and LDL-C levels in the ANV group were significantly higher than those in the EFV group, and the mean HDL-C level was significantly lower (Table 1). The proportion of patients with abnormal lipid profile was significantly higher in the ANV group than in the EFV group (Table 1); specifically, 24.4% of patients in the ANV group and 14.7% in the EFV group had abnormal TC (*P* = 0.002), 53.8% vs 39.3% for TG (*P* < 0.001), 46.2% vs 33.0% for HDL-C (*P* = 0.001), and 20.2% vs 12.4% for LDL-C (*P* = 0.006). This was consistent with some patients who had received the EFV-based regimen then switched to the ANV-based regimen due to dyslipidemia in real world.

**Fig 1:**
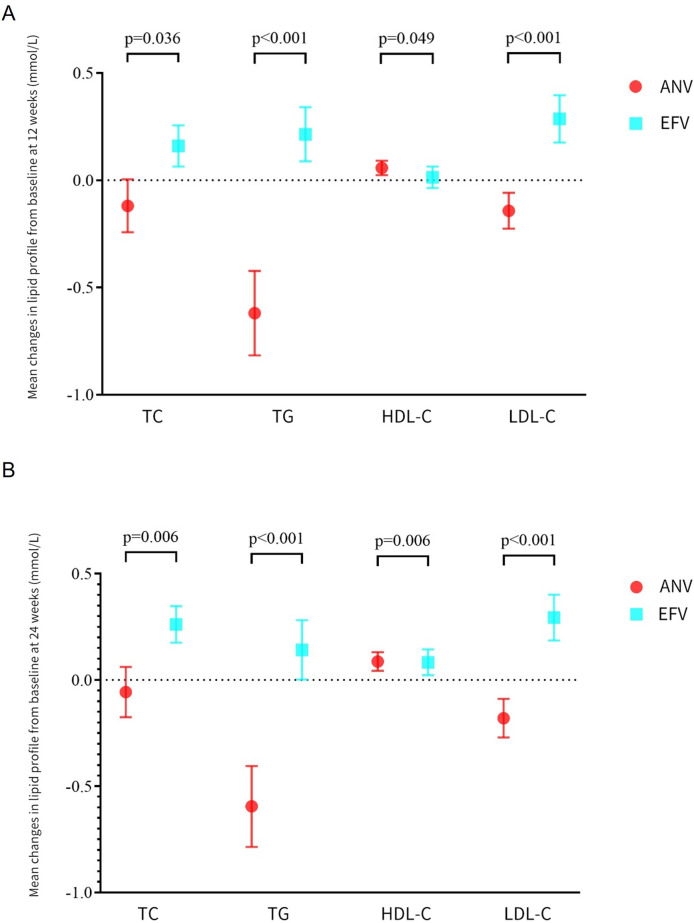
Mean changes from baseline in the lipid profile at week 12 (**A**) and week 24 (**B**) in all patients. The results were obtained through weighted analysis of propensity scores. Analysis of covariance was used for intergroup comparisons. At week 12, the *P* values for intragroup comparison of changes in lipids in the ANV and EFV groups were 0.057 and 0.001 for TC, *P* < 0.001 and *P* < 0.001 for TG, *P* < 0.001 and *P* = 0.574 for HDL-C, and *P* < 0.001 and *P* < 0.001 for LDL-C, respectively. At week 24, the *P* values for intragroup comparison of changes in lipids in the ANV and EFV groups were 0.341 and <0.001 for TC, *P* < 0.001 and *P* = 0.047 for TG, *P* < 0.001 and *P* = 0.008 for HDL-C, and *P* < 0.001 and *P* < 0.001 for LDL-C, respectively. ANV, ainuovirine; EFV, efavirenz; HDL-C, high density lipoprotein cholesterol; LDL-C, low density lipoprotein cholesterol; TC, total cholesterol; TG, triglyceride.

Considering the baseline lipid profile imbalances between the treatment groups, we used analysis of covariance to adjust each patient’s follow-up lipid levels for his or her baseline level, with the advantage of being unaffected by baseline differences. By using analysis of covariance, the baseline imbalances between groups were eliminated. Table 3 summarizes the mean changes from baseline in the lipid profile at week 24 by treatment and subgroup. In all patients, the differences in mean changes in the four lipid parameters between the ANV and EFV groups were statistically significant (*P* < 0.05, Fig. 1). By week 24, the mean (95% CI) changes in TC were −0.06 mmol/L (−0.17 to 0.06) for ANV and 0.26 mmol/L (0.18 to 0.35) for EFV (*P* = 0.006). TG levels were decreased with ANV (−0.60 mmol/L; 95% CI, −0.79 to −0.40) and increased with EFV (0.14 mmol/L; 95% CI, 0.00 to 0.28; *P* < 0.001). The increases in HDL-C with ANV (0.09 mmol/L; 95% CI, 0.04 to 0.13) were significantly greater than those with EFV (0.08 mmol/L; 95% CI, 0.02 to 0.14; *P* = 0.006). The LDL-C decrease was −0.18 mmol/L (−0.27 to −0.09) for ANV, compared with an increase of 0.29 mmol/L (0.19 to 0.40) for EFV (*P* < 0.001). Log (TG/HDL-C) was decreased from baseline both in the ANV (−0.11; 95% CI, −0.15 to −0.07) and EFV (−0.01; 95% CI, −0.03 to 0.02) groups, with no significant differences between groups (*P* = 0.063).

**TABLE 3:** Changes from baseline in the lipid profile at week 24 by treatment and subgroup^*a*^

	ANV group mean (95% CI)	EFV group mean (95% CI)	Treatment differences (95% CI)	*P* value
Overall patients	*n* = 318	*n* = 460		
TC	−0.06 (−0.17 to 0.06)	0.26 (0.18–0.35)	−0.17 (−0.29 to −0.05)	0.006
TG	−0.60 (−0.79 to −0.40)	0.14 (0.00–0.28)	−0.33 (−0.52 to −0.15)	<0.001
HDL-C	0.09 (0.04–0.13)	0.08 (0.02–0.14)	−0.08 (−0.14 to −0.02)	0.006
LDL-C	−0.18 (−0.27 to −0.09)	0.29 (0.19–0.40)	−0.29 (−0.41 to −0.17)	<0.001
Log (TG/HDL-C)	−0.11 (−0.15 to −0.07)	−0.01 (−0.03 to 0.02)	−0.04 (−0.07 to 0.00)	0.063
Dyslipidemia at baseline subgroup (A)^*b*^	*n* = 243	*n* = 281		
TC	−0.08 (−0.22 to 0.06)	0.20 (0.08–0.33)	−0.19 (−0.34 to −0.03)	0.020
TG	−0.82 (−1.06 to −0.58)	0.03 (−0.18 to 0.25)	−0.43 (−0.69 to −0.17)	0.001
HDL-C	0.10 (0.06–0.15)	0.15 (0.09–0.22)	−0.10 (−0.17 to −0.02)	0.009
LDL-C	−0.18 (−0.30 to −0.07)	0.26 (0.12–0.40)	−0.30 (−0.45 to −0.16)	<0.001
Log (TG/HDL-C)	−0.15 (−0.19 to −0.11)	−0.06 (−0.10 to −0.03)	−0.03 (−0.08 to 0.01)	0.162
Never taken lipid-lowering drugs subgroup (B)^*c*^	*n* = 260	*n* = 347		
TC	−0.10 (−0.22 to 0.03)	0.26 (0.16–0.36)	−0.14 (−0.27 to −0.00)	0.044
TG	−0.67 (−0.88 to −0.45)	0.12 (−0.00 to 0.24)	−0.25 (−0.42 to −0.08)	0.005
HDL-C	0.10 (0.05–0.14)	0.15 (0.09–0.21)	−0.09 (−0.16 to −0.02)	0.010
LDL-C	−0.21 (−0.31 to −0.11)	0.24 (0.12–0.36)	−0.22 (−0.35 to −0.09)	<0.001
Log (TG/HDL-C)	−0.13 (−0.17 to −0.10)	−0.03 (−0.06 to −0.00)	−0.03 (−0.07 to 0.01)	0.126
Dyslipidemia at baseline and never taken lipid-lowering drugs subgroup (C)	*n* = 197	*n* = 206		
TC	−0.12 (−0.27 to 0.03)	0.17 (0.01–0.32)	−0.10 (−0.27 to 0.08)	0.274
TG	−0.91 (−1.19 to −0.63)	−0.03 (−0.20 to 0.15)	−0.28 (−0.52 to −0.04)	0.021
HDL-C	0.11 (0.07–0.15)	0.21 (0.12–0.29)	−0.11 (−0.19 to −0.02)	0.012
LDL-C	−0.21 (−0.33 to −0.09)	0.16 (0.01–0.31)	−0.18 (−0.33 to −0.03)	0.019
Log (TG/HDL-C)	−0.18 (−0.22 to −0.14)	−0.10 (−0.13 to −0.06)	−0.02 (−0.07 to 0.02)	0.304

^*a*^ ANV, ainuovirine; CI, confidence interval; EFV, efavirenz; HDL-C, high density lipoprotein cholesterol; LDL-C, low density lipoprotein cholesterol; TC, total cholesterol; TG, triglyceride. Patient numbers were unweighted, and the remaining results were obtained through weighted analysis of propensity scores. Analysis of covariance was used for intergroup comparisons.

^*b*^ Dyslipidemia is defined as TC ≥5.2 mmol/L or TG ≥1.7 mmol/L or HDL-C＜1.0 mmol/L or LDL-C ≥3.4 mmol/L.

^*c*^ Patients who reported no use of lipid-lowering drugs at baseline, week 12, and week 24 were considered to have never taken lipid-lowering drugs.

**Fig 2:**
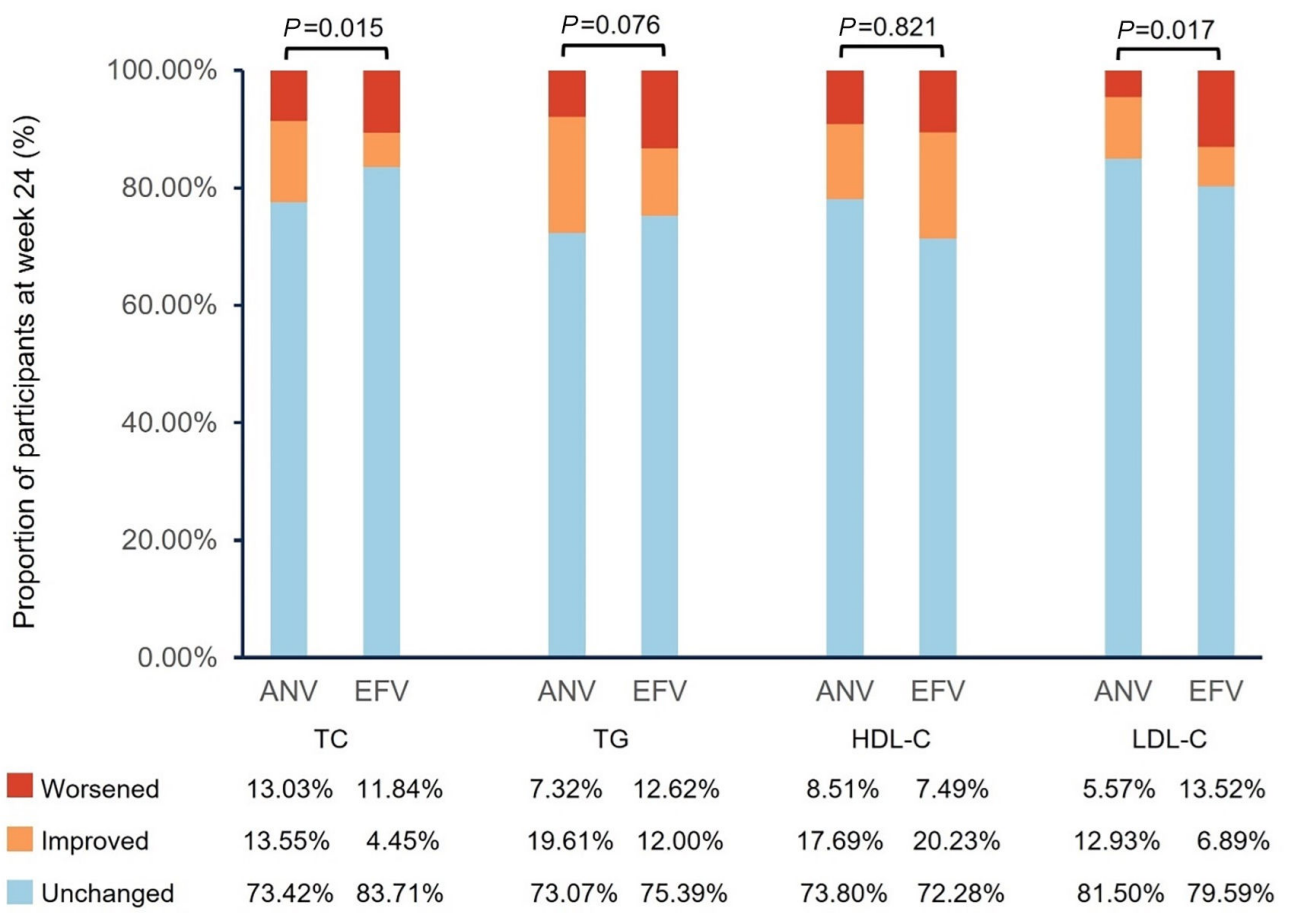
Proportion of patients with worsened, improved, and unchanged lipid profiles at week 24 among all patients. The results were obtained through weighted analysis of propensity scores. In the analyses, worsening was defined as the lipid level changing from normal at baseline to abnormal; improvement was defined as the lipid level changing from abnormal at baseline to normal; and the lipid level remaining normal or abnormal was defined as unchanged. ANV, ainuovirine; EFV, efavirenz; HDL-C, high density lipoprotein cholesterol; LDL-C, low density lipoprotein cholesterol; TC, total cholesterol; TG, triglyceride.

Figure 2 presents the proportion of patients with worsened, improved, and unchanged lipid profiles from baseline at week 24 among all patients. A higher proportion of patients in the ANV group had improved TC (13.55% vs 4.45%, *P* = 0.015) or LDL-C (12.93% vs 6.89%, *P* = 0.017) levels at week 24 than those in the EFV group. A numerically higher proportion of patients had their TG (19.61% vs 12.0%, *P* = 0.076) improved with ANV than with EFV, although with no significant differences. The proportion of patients with improved HDL-C was numerically lower in the ANV group than in the EFV group (17.69% vs 20.23%), with no significant differences between groups (*P* = 0.821). The results were similar at week 12, except with no significant differences in the proportion of patients with improved TC between the ANV and EFV group (13.85% vs 5.83%, *P* = 0.054) (Fig. S1).

Patients were stratified according to whether they had dyslipidemia at baseline or whether they were using lipid-lowering drugs and were divided into three subgroups: dyslipidemia at baseline (subgroup A), never taken lipid-lowering drugs (subgroup B), and dyslipidemia at baseline and never taken lipid-lowering drugs (subgroup C). In subgroup analyses, the mean changes in TC, TG, LDL-C, and log (TG/HDL-C) were more favorable with ANV than with EFV at week 24 for each of the three subgroups, although the mean changes in HDL-C favored EFV (Table 3). In subgroup A of dyslipidemia at baseline, the mean (95% CI) changes in TC were −0.08 mmol/L (−0.22 to 0.06) for ANV and 0.20 mmol/L (0.08 to 0.33) for EFV (*P* = 0.02). The decrease in TG was −0.82 mmol/L (−1.06 to −0.58) with ANV, compared with an increase of 0.03 mmol/L (−0.18 to 0.25) with EFV (*P* = 0.001). The increases in HDL-C levels were 0.10 mmol/L (0.06 to 0.15) with ANV and 0.15 mmol/L (0.09 to 0.22) with EFV (*P* = 0.009). LDL-C levels decreased with ANV (−0.18 mmol/L; 95% CI, −0.30 to −0.07) but increased with EFV (0.26 mmol/L; 95% CI, 0.12 to 0.40; *P* < 0.001). The decreases in log (TG/HDL-C) were −0.15 (−0.19 to −0.11) with ANV and −0.06 (−0.10 to −0.03) with EFV, with no significant differences (*P* = 0.162) between the two treatment groups. In subgroups B and C, which excluded patients on lipid-lowering medications to rule out the effects of lipid-lowering drugs on lipids, similar changes in lipid profiles were observed as in subgroup A, except that there were no significant differences (*P* = 0.274) between treatment groups for the mean changes in TC in subgroup C (Table 3).

### BMI changes

At week 24, there were no significant differences in mean (SD) BMI compared to baseline in either the ANV (*P* = 0.891) or EFV group (*P* = 0.698), and there were no significant differences in BMI distribution for either group (Table S3). The same results were observed at week 12 (Table S3). These data showed that the ANV-based regimen had no significant effect on body weight (BMI).

## DISCUSSION

To the best of our knowledge, this is the first real-world study to compare the efficacy and safety of switching to ANV from EFV with continuing EFV in treatment-experienced people with HIV-1. Our findings indicate that switching to ANV-based regimen resulted in considerable efficacy and favorable changes in lipid profile.

Throughout the 24-week study period, participants who switched to ANV-based regimen showed maintenance of virologic suppression, with no significant differences compared with the EFV group. Immune restoration, as measured by median changes from baseline in CD4^+^ T-cell counts and CD4^+^/CD8^+^ ratio, was similar between the ANV and EFV groups. The efficacy of ANV was comparable to that of EFV, which was consistent with the results of a previous randomized phase 3 trial (25).

Many antiretroviral regimens, including EFV, are related to adverse effects in serum lipids. This often necessitates lipid-lowering treatment (28, 29). Multiple studies have shown that EFV demonstrate more negative changes in TC, TG, and LDL-C than elvitegravir (EVG) (30–32), raltegravir (RAL) (33, 34), dolutegravir (DTG) (35), nevirapine (NVP) (36), and rilpivirine (RPV) (37) when used in people with HIV-1. According to the DRIVE-AHEAD trial, the EFV group exhibited higher mean increases in fasting LDL-C and non-HDL-C at week 96 compared with week 48 (38, 39), suggesting that the chance of developing dyslipidemia increases with prolonged use of EFV. In line with the previous studies, our data showed that participants who continued the EFV-based regimen for 24 weeks exhibited significant increases in TC, TG, and LDL-C (Fig. 1B).

The association of adverse impact on serum lipids with certain antiretroviral therapies has prompted a strategy of switching the potentially offending agent for an alternative regimen. According to the European AIDS Clinical Society (EACS) guideline 2022, in the event of adverse effects, it is recommended to switch within the same drug class if the alternative has equal potency and there is no evidence of resistance (40). Recent studies indicated that newer NNRTIs, such as doravirine (DOR), have more favorable impact on lipids (39, 41). Other studies on switching therapy have found that switching to DOR demonstrates favorable changes in lipid profiles. In the DRIVE-SHIFT Trial, participants who switched to DOR-based regimen from ritonavir-boosted protease inhibitor had significantly greater reductions in fasting LDL-C and non-HDL-C at week 24 (42). A retrospective multi-center Italian study demonstrated a significant reduction in both cholesterol and triglyceride levels 24 weeks after switching to DOR-containing/-based regimens (43). In the present study, patients who switched to ANV, another novel NNRTI, showed decreases in mean TC, TG, and LDL-C both at weeks 12 and 24. In contrast, patients continuing with EFV experienced increases in these lipid parameters (Fig. 1). Subgroup analyses confirmed that after ruling out the confounding effects of baseline lipid levels and the use of lipid-lowering drugs, ANV was still superior to EFV for changes in TC, TG, and LDL-C (Table 3). The atherogenic index of plasma (AIP), one of the predictors for coronary atherosclerosis risk, is calculated as Log (TG/HDL-C) (44). Log (TG/HDL-C) at baseline was higher in the ANV group than in the EFV group (Table 1) and decreased numerically more with ANV than with EFV at week 24 (Table 3). Similar results were observed in all three subgroups. Moreover, a significantly higher proportion of patients had improved TC and LDL-C in the ANV group than in the EFV group at week 24 (Fig. 2). Since higher LDL-C levels are associated with an increased risk of CVD and “lower is better for longer” (45) the favorable changes in LDL-C in participants who switched to ANV from EFV could have a beneficial effect on CVD prevention.

Weight gain after ART initiation is common in people with HIV (46). A pool analysis of 8 randomized controlled clinical trials, including 5,680 people with HIV-initiating ART between 2003 and 2015, reported that 48.6%, 36.6%, and 17.3% of participants had a weight gain of 3%, 5%, and 10% from baseline, respectively (47). And the weight gain was greater among those taking integrase strand transfer inhibitors (INSTIs) than NNRTIs and PIs (47). This study found that ANV had little effect on body weight (BMI) and would not cause weight gain.

Several limitations to our study should be noted. The first is the limitation inherent to retrospective analysis in real clinical setting, including the risk of confounding bias and selection bias, which were inevitable. Since patients were not randomly assigned, decisions of switching from EFV to ANV were made upon the attending physician’s discretion and potentially had a risk of confounding variables. Furthermore, the follow-up period was only 24 weeks, which could be inadequate to determine the effectiveness of switching to ANV, as improvement in lipid profile and CVD risk need long-term evaluation. We will retain the cohort for long-term follow-up, and the results of 48 weeks or even longer will be presented later. Third, there may be individual disparities in atherosclerotic cardiovascular disease (ASCVD) risk score at baseline between the two groups, which could influence the outcomes but were not stratified in the current analysis. However, propensity score weighting using overlap weights was used to overcome this limitation by balancing the baseline characteristics between the two groups. Nevertheless, these findings should be interpreted with caution. Further randomized controlled trials with CVD risk stratification and longer follow-up to verify the lipid-friendly properties of ANV are needed, and the mechanisms of newer NNRTIs on lipid metabolism require further exploration (48).

### Conclusions

This retrospective study suggested that in this scenario, ANV-based regimen showed comparable virologic/immunologic effect compared with EFV-based regimen. ANV exhibited favorable changes in lipid profiles in participants who switched from EFV. In addition, ANV did not cause weight gain. Overall, ANV is a promising switching option for patients who may have other risk factors for metabolic syndrome or cardiovascular diseases.

## SUPPLEMENTAL MATERIAL

Supplemental tables and figure aac.01668-23-s0001.docx Table S1 to S3, Fig. S1

ASM does not own the copyrights to Supplemental Material that may be linked to, or accessed through, an article. The authors have granted ASM a non-exclusive, world-wide license to publish the Supplemental Material files. Please contact the corresponding author directly for reuse.

## References

[1] Mocroft A, Ledergerber B, Katlama C, Kirk O, Reiss P, d’Arminio Monforte A, Knysz B, Dietrich M, Phillips AN, Lundgren JD, EuroSIDA study group. 2003. Decline in the AIDS and death rates in the EuroSIDA study: an observational study. Lancet 362:22–29. doi:10.1016/s0140-6736(03)13802-0 12853195

[2] Bhaskaran K, Hamouda O, Sannes M, Boufassa F, Johnson AM, Lambert PC, Porter K, CASCADE Collaboration. 2008. Changes in the risk of death after HIV seroconversion compared with mortality in the general population. JAMA 300:51–59. doi:10.1001/jama.300.1.51 18594040

[3] Palella FJ, Baker RK, Moorman AC, Chmiel JS, Wood KC, Brooks JT, Holmberg SD, HIV Outpatient Study Investigators. 2006. Mortality in the highly active antiretroviral therapy era: changing causes of death and disease in the HIV outpatient study. J Acquir Immune Defic Syndr 43:27–34. doi:10.1097/01.qai.0000233310.90484.16 16878047

[4] Croxford S, Kitching A, Desai S, Kall M, Edelstein M, Skingsley A, Burns F, Copas A, Brown AE, Sullivan AK, Delpech V. 2017. Mortality and causes of death in people diagnosed with HIV in the era of highly active antiretroviral therapy compared with the general population: an analysis of a national observational cohort. Lancet Public Health 2:e35–e46. doi:10.1016/S2468-2667(16)30020-2 29249478

[5] Feinstein MJ, Bahiru E, Achenbach C, Longenecker CT, Hsue P, So-Armah K, Freiberg MS, Lloyd-Jones DM. 2016. Patterns of cardiovascular mortality for HIV-infected adults in the United States: 1999 to 2013. Am J Cardiol 117:214–220. doi:10.1016/j.amjcard.2015.10.030 26639041 PMC5308060

[6] Shah ASV, Stelzle D, Lee KK, Beck EJ, Alam S, Clifford S, Longenecker CT, Strachan F, Bagchi S, Whiteley W, Rajagopalan S, Kottilil S, Nair H, Newby DE, McAllister DA, Mills NL. 2018. Global burden of atherosclerotic cardiovascular disease in people living with HIV: systematic review and meta-analysis. Circulation 138:1100–1112. doi:10.1161/CIRCULATIONAHA.117.033369 29967196 PMC6221183

[7] Nsagha DS, Assob JCN, Njunda AL, Tanue EA, Kibu OD, Ayima CW, Ngowe MN. 2015. Risk factors of cardiovascular diseases in HIV/AIDS patients on HAART. Open AIDS J 9:51–59. doi:10.2174/1874613601509010051 26587072 PMC4645867

[8] Lo J. 2011. Dyslipidemia and lipid management in HIV-infected patients. Curr Opin Endocrinol Diabetes Obes 18:144–147. doi:10.1097/MED.0b013e328344556e 21297466 PMC3154840

[9] Riddler SA, Smit E, Cole SR, Li R, Chmiel JS, Dobs A, Palella F, Visscher B, Evans R, Kingsley LA. 2003. Impact of HIV infection and HAART on serum lipids in men. JAMA 289:2978–2982. doi:10.1001/jama.289.22.2978 12799406

[10] Funderburg NT, Mehta NN. 2016. Lipid abnormalities and inflammation in HIV infection. Curr HIV/AIDS Rep 13:218–225. doi:10.1007/s11904-016-0321-0 27245605 PMC4977198

[11] Fontas E, van Leth F, Sabin CA, Friis-Møller N, Rickenbach M, d’Arminio Monforte A, Kirk O, Dupon M, Morfeldt L, Mateu S, Petoumenos K, El-Sadr W, de Wit S, Lundgren JD, Pradier C, Reiss P, D:A:D Study Group. 2004. Lipid profiles in HIV-infected patients receiving combination antiretroviral therapy: are different antiretroviral drugs associated with different lipid profiles? J Infect Dis 189:1056–1074. doi:10.1086/381783 14999610

[12] Friis-Møller N, Reiss P, Sabin CA, Weber R, Monforte Ad, El-Sadr W, Thiébaut R, De Wit S, Kirk O, Fontas E, Law MG, Phillips A, Lundgren JD, DAD Study Group. 2007. Class of antiretroviral drugs and the risk of myocardial infarction. N Engl J Med 356:1723–1735. doi:10.1056/NEJMoa062744 17460226

[13] Pefura Yone EW, Betyoumin AF, Kengne AP, Kaze Folefack FJ, Ngogang J. 2011. First-line antiretroviral therapy and dyslipidemia in people living with HIV-1 in Cameroon: a cross-sectional study. AIDS Res Ther 8:33. doi:10.1186/1742-6405-8-33 21943115 PMC3197472

[14] Souza SJ, Luzia LA, Santos SS, Rondó PHC. 2013. Lipid profile of HIV-infected patients in relation to antiretroviral therapy: a review. Rev Assoc Med Bras (1992) 59:186–198. doi:10.1016/j.ramb.2012.11.003 23582562

[15] Liu S, Wei B, Liang W, Chen T, Deng L, Zhao M, Wan J. 2023. The effects of ART on the dynamics of lipid profiles in Chinese Han HIV-infected patients: comparison between NRTI/NNRTI and NRTI/INSTI. Front Public Health 11:1161503. doi:10.3389/fpubh.2023.1161503 37181701 PMC10174832

[16] Currier JS, Lundgren JD, Carr A, Klein D, Sabin CA, Sax PE, Schouten JT, Smieja M, Working Group 2. 2008. Epidemiological evidence for cardiovascular disease in HIV-infected patients and relationship to highly active antiretroviral therapy. Circulation 118:e29–e35. doi:10.1161/CIRCULATIONAHA.107.189624 18566319 PMC5153327

[17] Hedayatnia M, Asadi Z, Zare-Feyzabadi R, Yaghooti-Khorasani M, Ghazizadeh H, Ghaffarian-Zirak R, Nosrati-Tirkani A, Mohammadi-Bajgiran M, Rohban M, Sadabadi F, Rahimi H-R, Ghalandari M, Ghaffari M-S, Yousefi A, Pouresmaeili E, Besharatlou M-R, Moohebati M, Ferns GA, Esmaily H, Ghayour-Mobarhan M. 2020. Dyslipidemia and cardiovascular disease risk among the MASHAD study population. Lipids Health Dis 19:42. doi:10.1186/s12944-020-01204-y 32178672 PMC7075010

[18] Ference BA, Ginsberg HN, Graham I, Ray KK, Packard CJ, Bruckert E, Hegele RA, Krauss RM, Raal FJ, Schunkert H, et al.. 2017. Low-density lipoproteins cause atherosclerotic cardiovascular disease. 1. Evidence from genetic, epidemiologic, and clinical studies. A consensus statement from the European Atherosclerosis Society Consensus Panel. Eur Heart J 38:2459–2472. doi:10.1093/eurheartj/ehx144 28444290 PMC5837225

[19] AIDS Panel in Tropical diseases and Parasitology Branch of Chinese Medical Association. 2023. Chinese expert consensus on integrated lipid management in HIV/AIDS (in Chinese). Chin J Intern Med 62:661–672. doi:10.3760/cma.j.cn112138-20230321-00165 37263949

[20] Brunzell JD, Ayyobi AF. 2003. Dyslipidemia in the metabolic syndrome and type 2 diabetes mellitus. Am J Med 115:24S–28S. doi:10.1016/j.amjmed.2003.08.011 14678862

[21] Kryst J, Kawalec P, Pilc A. 2015. Efavirenz-based regimens in antiretroviral-naive HIV-infected patients: a systematic review and meta-analysis of randomized controlled trials. PLoS One 10:e0124279. doi:10.1371/journal.pone.0124279 25933004 PMC4416921

[22] 2016. Consolidated guidelines on the use of antiretroviral drugs for treating and preventing HIV infection: recommendations for a public health approach. 2nd ed. World Health Organization, Geneva. https://www.who.int/publications/i/item/9789241549684. 27466667

[23] Center for STD/AIDS Prevention and Control, Chinese Center for Disease Control and Prevention. 2016. Manual of national free antiviral drug for AIDS treatment (in Chinese). 4th ed. People’s Medical Publishing House, Beijing.

[24] Sun L-Q, Liu J-Y, He Y, Zhou Y, Xu L-M, Zhang L-K, Zhao F, Liu X-N, Song Y, Cao T-Z, Tian Y-M, Rao M, Wang H. 2020. Evolution of blood lipids and risk factors of dyslipidemia among people living with human immunodeficiency virus who had received first-line antiretroviral regimens for 3 years in Shenzhen. Chin Med J (Engl) 133:2808–2815. doi:10.1097/CM9.0000000000001245 33273329 PMC10631593

[25] Su B, Gao G, Wang M, Lu Y, Li L, Chen C, Chen Y, Song C, Yu F, Li Y, et al.. 2023. Efficacy and safety of ainuovirine versus efavirenz combination therapies with lamivudine/tenofovir disoproxil fumarate for medication of treatment-naïve HIV-1-positive adults: week 48 results of a randomized controlled phase 3 clinical trial followed by an open-label setting until week 96. Lancet Reg Health West Pac 36:100769. doi:10.1016/j.lanwpc.2023.100769 37547039 PMC10398592

[26] Joint Committee on the Chinese Guidelines for Lipid Management. 2023. Chinese guidelines for lipid management (2023). Zhonghua Xin Xue Guan Bing Za Zhi 51:221–255. doi:10.3760/cma.j.cn112148-20230119-00038 36925135

[27] Mlcoch T, Hrnciarova T, Tuzil J, Zadak J, Marian M, Dolezal T. 2019. Propensity score weighting using overlap weights: a new method applied to regorafenib clinical data and a cost-effectiveness analysis. Value Health 22:1370–1377. doi:10.1016/j.jval.2019.06.010 31806193

[28] Tashima KT, Bausserman L, Alt EN, Aznar E, Flanigan TP. 2003. Lipid changes in patients initiating efavirenz- and indinavir-based antiretroviral regimens. HIV Clin Trials 4:29–36. doi:10.1310/f2v7-3r46-vx6j-241r 12577194

[29] Gerber JG, Rosenkranz SL, Fichtenbaum CJ, Vega JM, Yang A, Alston BL, Brobst SW, Segal Y, Aberg JA, AIDS Clinical Trials Group A5108 Team. 2005. Effect of efavirenz on the pharmacokinetics of simvastatin, atorvastatin, and pravastatin: results of AIDS Clinical Trials Group 5108 Study. J Acquir Immune Defic Syndr 39:307–312. doi:10.1097/01.qai.0000167156.44980.33 15980690

[30] Sax PE, DeJesus E, Mills A, Zolopa A, Cohen C, Wohl D, Gallant JE, Liu HC, Zhong L, Yale K, White K, Kearney BP, Szwarcberg J, Quirk E, Cheng AK, GS-US-236-0102 study team. 2012. Co-formulated elvitegravir, cobicistat, emtricitabine, and tenofovir versus co-formulated efavirenz, emtricitabine, and tenofovir for initial treatment of HIV-1 infection: a randomised, double-blind, phase 3 trial, analysis of results after 48 weeks. Lancet 379:2439–2448. doi:10.1016/S0140-6736(12)60917-9 22748591

[31] Zolopa A, Gallant J, Cohen C, Sax P, Dejesus E, Mills A, Wohl D, Liu H, Rhee M, Szwarcberg J. 2012. Elvitegravir/cobicistat/emtricitabine/tenofovir DF (Quad) has durable efficacy and differentiated safety compared to efavirenz/emtricitabine/tenofovir DF at week 96 in treatment-naïve HIV-1-infected patients. J Int AIDS Soc 15:18219. doi:10.7448/IAS.15.6.18219

[32] Wohl DA, Cohen C, Gallant JE, Mills A, Sax PE, Dejesus E, Zolopa A, Liu HC, Plummer A, White KL, Cheng AK, Rhee MS, Szwarcberg J, GS-US-236-0102 Study Team. 2014. A randomized, double-blind comparison of single-tablet regimen elvitegravir/cobicistat/emtricitabine/tenofovir DF versus single-tablet regimen efavirenz/emtricitabine/tenofovir DF for initial treatment of HIV-1 infection: analysis of week 144 results. J Acquir Immune Defic Syndr 65:e118–e120. doi:10.1097/QAI.0000000000000057 24256630

[33] Lennox JL, DeJesus E, Lazzarin A, Pollard RB, Madruga JVR, Berger DS, Zhao J, Xu X, Williams-Diaz A, Rodgers AJ, Barnard RJO, Miller MD, DiNubile MJ, Nguyen B-Y, Leavitt R, Sklar P, STARTMRK investigators. 2009. Safety and efficacy of raltegravir-based versus efavirenz-based combination therapy in treatment-naive patients with HIV-1 infection: a multicentre, double-blind randomised controlled trial. Lancet 374:796–806. doi:10.1016/S0140-6736(09)60918-1 19647866

[34] Lennox JL, Dejesus E, Berger DS, Lazzarin A, Pollard RB, Ramalho Madruga JV, Zhao J, Wan H, Gilbert CL, Teppler H, Rodgers AJ, Barnard RJO, Miller MD, Dinubile MJ, Nguyen B-Y, Leavitt R, Sklar P, STARTMRK Investigators. 2010. Raltegravir versus efavirenz regimens in treatment-naive HIV-1-infected patients: 96-week efficacy, durability, subgroup, safety, and metabolic analyses. J Acquir Immune Defic Syndr 55:39–48. doi:10.1097/QAI.0b013e3181da1287 20404738 PMC6065510

[35] van Lunzen J, Maggiolo F, Arribas JR, Rakhmanova A, Yeni P, Young B, Rockstroh JK, Almond S, Song I, Brothers C, Min S. 2012. Once daily dolutegravir (S/GSK1349572) in combination therapy in antiretroviral-naive adults with HIV: planned interim 48 week results from SPRING-1, a dose-ranging, randomised, phase 2b trial. Lancet Infect Dis 12:111–118. doi:10.1016/S1473-3099(11)70290-0 22018760

[36] van Leth F, Phanuphak P, Stroes E, Gazzard B, Cahn P, Raffi F, Wood R, Bloch M, Katlama C, Kastelein JJP, Schechter M, Murphy RL, Horban A, Hall DB, Lange JMA, Reiss P. 2004. Nevirapine and efavirenz elicit different changes in lipid profiles in antiretroviral-therapy-naive patients infected with HIV-1. PLoS Med 1:e19. doi:10.1371/journal.pmed.0010019 15526045 PMC523838

[37] Tebas P, Sension M, Arribas J, Duiculescu D, Florence E, Hung C-C, Wilkin T, Vanveggel S, Stevens M, Deckx H, ECHO and THRIVE Study Groups. 2014. Lipid levels and changes in body fat distribution in treatment-naive, HIV-1-Infected adults treated with rilpivirine or Efavirenz for 96 weeks in the ECHO and THRIVE trials. Clin Infect Dis 59:425–434. doi:10.1093/cid/ciu234 24729492

[38] Orkin C, Squires KE, Molina J-M, Sax PE, Wong W-W, Sussmann O, Kaplan R, Lupinacci L, Rodgers A, Xu X, Lin G, Kumar S, Sklar P, Nguyen B-Y, Hanna GJ, Hwang C, Martin EA, DRIVE-AHEAD Study Group. 2019. Doravirine/lamivudine/tenofovir disoproxil fumarate is non-inferior to efavirenz/emtricitabine/tenofovir disoproxil fumarate in treatment-naive adults with human immunodeficiency virus-1 infection: week 48 results of the DRIVE-AHEAD trial. Clin Infect Dis 68:535–544. doi:10.1093/cid/ciy540 30184165 PMC6355823

[39] Orkin C, Squires KE, Molina J-M, Sax PE, Sussmann O, Lin G, Kumar S, Hanna GJ, Hwang C, Martin E, Teppler H. 2021. Doravirine/lamivudine/tenofovir disoproxil fumarate (TDF) versus efavirenz/emtricitabine/TDF in treatment-naive adults with human immunodeficiency virus type 1 infection: week 96 results of the randomized, double-blind, phase 3 DRIVE-AHEAD noninferiority trial. Clin Infect Dis 73:33–42. doi:10.1093/cid/ciaa822 33336698 PMC8246893

[40] EACS guidelines V11.1. 2022. European AIDS Clinical Society. Available from: https://www.eacsociety.org/media/guidelines-11.1_final_09-10.pdf. Retrieved 31 Oct 2023.

[41] Molina J-M, Squires K, Sax PE, Cahn P, Lombaard J, DeJesus E, Lai M-T, Rodgers A, Lupinacci L, Kumar S, Sklar P, Hanna GJ, Hwang C, Martin EA, DRIVE-FORWARD trial group. 2020. Doravirine versus ritonavir-boosted darunavir in antiretroviral-naive adults with HIV-1 (DRIVE-FORWARD): 96-week results of a randomised, double-blind, non-inferiority, phase 3 trial. Lancet HIV 7:e16–e26. doi:10.1016/S2352-3018(19)30336-4 31740348

[42] Johnson M, Kumar P, Molina J-M, Rizzardini G, Cahn P, Bickel M, Mallolas J, Zhou Y, Morais C, Kumar S, Sklar P, Hanna GJ, Hwang C, Greaves W, DRIVE-SHIFT Study Group. 2019. Switching to doravirine/lamivudine/tenofovir disoproxil fumarate (DOR/3TC/TDF) maintains HIV-1 virologic suppression through 48 weeks: results of the DRIVE-SHIFT trial. J Acquir Immune Defic Syndr 81:463–472. doi:10.1097/QAI.0000000000002056 30985556 PMC6905402

[43] Mazzitelli M, Antoni MD, Castelli F, Ripamonti D, Zuglian G, Lapadula G, Fabbiani M, Ferraresi A, Putaggio C, Cattelan AM, Quiros-Roldan E. 2022. Real-life use of doravirine in treatment-experienced people living with HIV: a multicenter Italian study. Medicine (Baltimore) 101:e29855. doi:10.1097/MD.0000000000029855 35905209 PMC9333545

[44] Dobiásová M, Frohlich J. 2001. The plasma parameter log (TG/HDL-C) as an atherogenic index: correlation with lipoprotein particle size and esterification rate inapob-lipoprotein-depleted plasma (FER_HDL_). Clin Biochem 34:583–588. doi:10.1016/s0009-9120(01)00263-6 11738396

[45] Penson PE, Pirro M, Banach M. 2020. LDL-C: lower is better for longer-even at low risk. BMC Med 18:320. doi:10.1186/s12916-020-01792-7 33032586 PMC7545575

[46] Koethe JR, Jenkins CA, Lau B, Shepherd BE, Justice AC, Tate JP, Buchacz K, Napravnik S, Mayor AM, Horberg MA, Blashill AJ, Willig A, Wester CW, Silverberg MJ, Gill J, Thorne JE, Klein M, Eron JJ, Kitahata MM, Sterling TR, Moore RD, North American AIDS Cohort Collaboration on Research and Design (NA-ACCORD). 2016. Rising obesity prevalence and weight gain among adults starting antiretroviral therapy in the United States and Canada. AIDS Res Hum Retroviruses 32:50–58. doi:10.1089/aid.2015.0147 26352511 PMC4692122

[47] Sax PE, Erlandson KM, Lake JE, Mccomsey GA, Orkin C, Esser S, Brown TT, Rockstroh JK, Wei X, Carter CC, Zhong L, Brainard DM, Melbourne K, Das M, Stellbrink H-J, Post FA, Waters L, Koethe JR. 2020. Weight gain following initiation of antiretroviral therapy: risk factors in randomized comparative clinical trials. Clin Infect Dis 71:1379–1389. doi:10.1093/cid/ciz999 31606734 PMC7486849

[48] Curran A, Rull A, Navarro J, Vidal-González J, Martin-Castillo M, Burgos J, Falcó V, Ribera E, Torrella A, Planas B, Peraire J, Crespo M. 2020. Lipidomics reveals reduced inflammatory lipid species and storage lipids after switching from EFV/FTC/TDF to RPV/FTC/TDF: a randomized open-label trial. J Clin Med 9:1246. doi:10.3390/jcm9051246 32344934 PMC7288166

