# Supplementary material for: Efficacy and effect on lipid profiles of switching to ainuovirine-based regimen versus continuing efavirenz-based regimen in people with HIV-1: 24-week results from a real-world, retrospective, multi-center cohort study
Source: Antimicrob Agents Chemother. 2024 Mar 14;68(4):e01668-23. doi: 10.1128/aac.01668-23 (PMC10989015; doi:10.1128/aac.01668-23)
Supplement: Supplemental tables and figure — Table S1 to S3, Fig. S1 [file aac.01668-23-s0001.docx]

**Supplemental Table S1. Name of 7 clinical centers**

| No. | Institution | City |
| --- | --- | --- |
| 1 | Department of Dermatology, Shandong Provincial Public Health Clinical Center | Jinan |
| 2 | Department of Infection and Immunology with Chinese Integrative Medicine, Wuhan Jinyintan Hospital, Tongji Medical College of Huazhong University of Science and Technology | Wuhan |
| 3 | Infectious Disease Center, Guangzhou Eighth People's Hospital, Guangzhou Medical University | Guangzhou |
| 4 | Department of Infectious Disease, GuiYang Public Health Clinical Center | GuiYang |
| 5 | Department of Infection and Immunology, The First Hospital of Changsha City, Xiangya School of Medicine of Central South University | Changsha |
| 6 | Department of Outpatient, Yunnan Provincial Infectious Disease Hospital | Kunming |

**Supplemental Table S2. Definition of limit of quantification (LOQ) by six centers**

| No. | Institution | LOQ, copies/ml |
| --- | --- | --- |
| 1 | Department of Dermatology, Shandong Public Health Clinical Center | 20 |
| 2 | Department of Infection and Immunology with Chinese Integrative Medicine, Wuhan Jinyintan Hospital, Tongji Medical College of Huazhong University of Science and Technology | 100 |
| 3 | Infectious Disease Center, Guangzhou Eighth People's Hospital, Guangzhou Medical University | 20 |
| 4 | Department of Infectious Disease, GuiYang Public Health Clinical Center | 20 |
| 5 | Department of Infection and Immunology, The First Hospital of Changsha City, Xiangya School of Medicine of Central South University | 20 |
| 6 | Department of Outpatient, Yunnan Provincial Infectious Disease Hospital | 20 |

**Supplemental Fig. S1. Proportion of patients with worsened, improved, and unchanged lipid profile at week 12 in overall patients.** The results were obtained through weighted analysis on propensity score. In the analyses, worsen defined as the lipid level changed from normal at baseline to abnormal; improve defined as the lipid level changed from abnormal at baseline to normal; the lipid level remained normal or abnormal defined as unchanged. ANV, ainuovirine; EFV, efavirenz; HDL-C, high density lipoprotein cholesterol; LDL-C, low density lipoprotein cholesterol; TC, total cholesterol; TG, triglyceride.


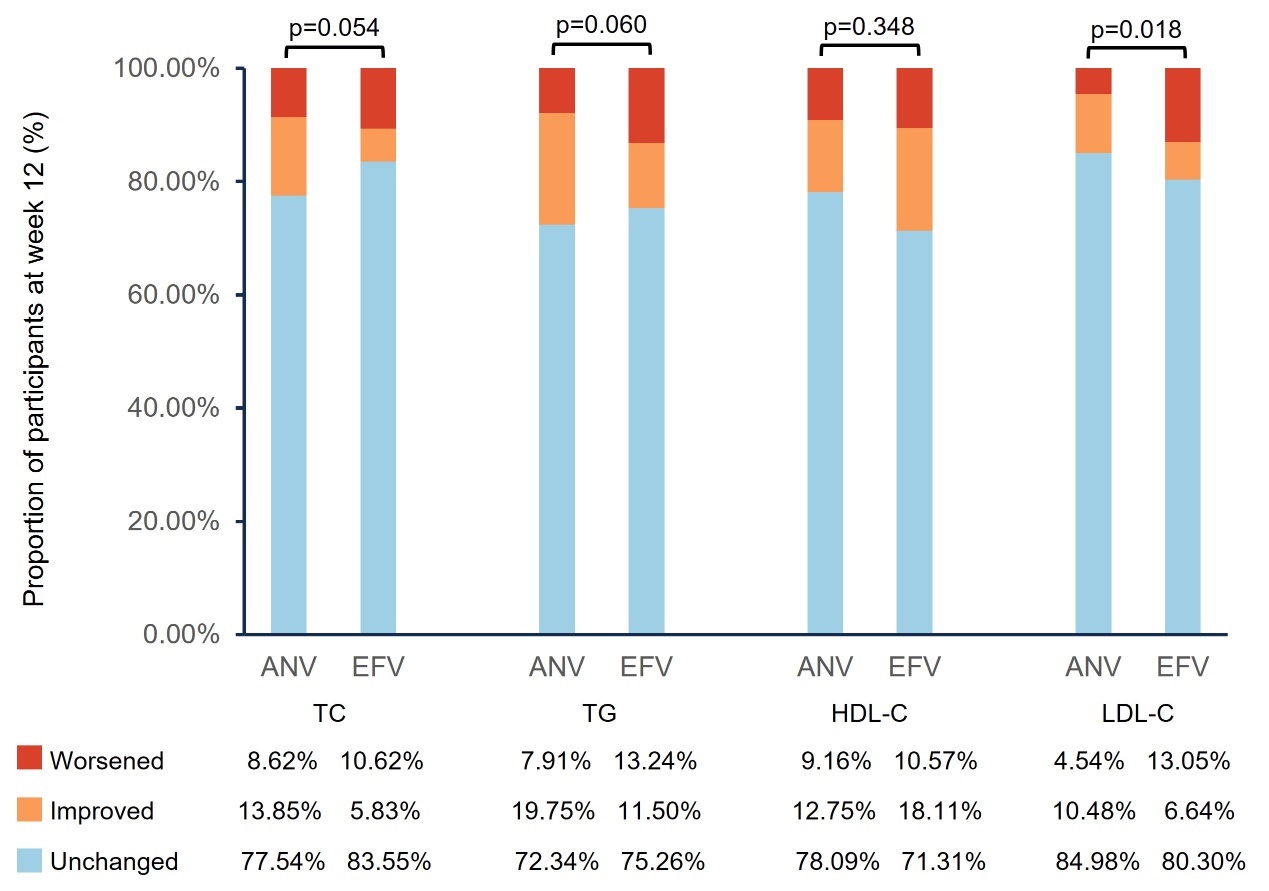


**Supplemental Table S3. BMI Outcomes at Week 12 and 24**

|  | ANV group | EFV group | p-value | SMD |
| --- | --- | --- | --- | --- |
| Week 12, mean (SD) | 23.01 (2.95)* | 22.59 (3.27)* | 0.105 | 0.134 |
| <18.5, n (%) | 10.8 (7.1) | 10.5 (7.3) | 0.798 | 0.083 |
| 18.5~24, n (%) | 86.7 (57.1) | 86.5 (60.7) |  |  |
| 24~28, n (%) | 48.9 (32.2) | 40.8 (28.6) |  |  |
| ≥28, n (%) | 5.5 (3.6) | 4.7 (3.3) |  |  |
| Week 24, mean (SD) | 22.85 (3.19)* | 22.73 (3.20)* | 0.689 | 0.037 |
| <18.5, n (%) | 9.9 (8.9) | 10.0 (7.1) | 0.641 | 0.115 |
| 18.5~24, n (%) | 65.0 (58.2) | 86.6 (61.0) |  |  |
| 24~28, n (%) | 30.9 (27.7) | 40.5 (28.5) |  |  |
| ≥28, n (%) | 5.8 (5.2) | 4.8 (3.4) |  |  |
| Paired Chi-square test (McNemar-Bowker test) was used for intragroup comparison between baseline and follow-up. ANV, Ainuovirine; EFV, Efavirenz; SD, standard deviation; SMD, standardized mean differences.  *P-values for intra-group comparison at week 12 and week 24 were 0.890 and 0.891 with ANV, and 0.467 and 0.698 with EFV, respectively. | | | | |
